# Comparative Transcriptome Analysis Identifies Putative Genes Involved in Steroid Biosynthesis in *Euphorbia tirucalli*

**DOI:** 10.3390/genes9010038

**Published:** 2018-01-15

**Authors:** Weibo Qiao, Changfu Li, Isidore Mosongo, Qin Liang, Mengdi Liu, Xin Wang

**Affiliations:** 1 CAS Key Laboratory of Plant Germplasm Enhancement and Specialty Agriculture, Wuhan Botanical Garden, Chinese Academy of Sciences, Wuhan 430074, China; qwb2013@163.com (W.Q.); lichangfu@wbgcas.cn (C.L.); isidoremosongo.im@gmail.com (I.M.); liangqin2017@163.com (Q.L.); liumengdi15@mails.ucas.edu.cn (M.L.); 2 University of Chinese Academy of Sciences, Beijing 100049, China

**Keywords:** *Euphorbia tirucalli*, oxidosqualene cyclase, transcriptome, steroid

## Abstract

Phytochemical analysis of different *Euphorbia tirucalli* tissues revealed a contrasting tissue-specificity for the biosynthesis of euphol and β-sitosterol, which represent the two pharmaceutically active steroids in *E. tirucalli*. To uncover the molecular mechanism underlying this tissue-specificity for phytochemicals, a comprehensive *E. tirucalli* transcriptome derived from its root, stem, leaf and latex was constructed, and a total of 91,619 unigenes were generated with 51.08% being successfully annotated against the non-redundant (Nr) protein database. A comparison of the transcriptome from different tissues discovered members of unigenes in the upstream steps of sterol backbone biosynthesis leading to this tissue-specific sterol biosynthesis. Among them, the putative oxidosqualene cyclase (OSC) encoding genes involved in euphol synthesis were notably identified, and their expressions were significantly up-regulated in the latex. In addition, genome-wide differentially expressed genes (DEGs) in the different *E. tirucalli* tissues were identified. The cluster analysis of those DEGs showed a unique expression pattern in the latex compared with other tissues. The DEGs identified in this study would enrich the insights of sterol biosynthesis and the regulation mechanism of this latex-specificity.

## 1. Introduction

Euphorbiaceae is a large plant family that includes more than 8000 species, many of which synthesize milky latex in a specialized structure called laticier [1]. The *Euphorbia tirucalli* plant, belonging to this group of latex-producing species, is native to the temperate regions of the world such as East Africa [2]. So far, the latex of *E. tirucalli* has been used in traditional folk medicine to treat sexual impotence, warts, epilepsy, toothache, hemorrhoids, and snake bites in many African countries [3]. In addition, extracts from the latex or stem of *E. tirucalli* have also been confirmed to have a variety of pharmacological effects, such as oxytoxic, antiarthritic, antiviral, antimicrobial and anti-inflammatory activities [4,5,6,7,8]. 

Phytochemical studies indicate that the occurrences of steroids or triterpenoids in the *E. tirucalli* latex confer those medicinal effects [9,10,11]. For instance, euphol, the most abundant steroid in the latex of *E. tirucalli*, can selectively induce human gastric cancer cells apoptosis and inhibit cancer cell development [12]. Besides, the *E. tirucalli* plant accumulates an array of steroids or terpenoids whose structures are similar to diesel oils, making it an ideal biofuel resource [13].

In general, the biosynthesis of steroids is derived from the isoprenoid pathway through the C5 unit (isopentenyl diphosphate (IPP) or dimethylallyl diphosphate (DMAPP)), which stems from either the mevalonate (MVA) or the methylerythritol 4-phosphate (MEP) pathway. In the phytosterol biosynthetic pathway, both IPP and DMAPP are condensed to geranyl diphosphate (C_10_, GPP) by the action of geranyl diphosphate synthase (GPPS); then, a farnesyl diphosphate synthase (FPPS) catalyzes the condensation of IPP with GPP to yield farnesyl diphosphate (C_15_, FPP). Next, two molecules of FPP are converted into squalene by squalene synthase (SQS), and this step is followed by the C2–C3 epoxidization of squalene by squalene epoxidase (SE) to generate 2,3-oxidosqualene. Thereafter, the scaffold of phytosterols is formed by cyclization of 2,3-oxidosqualene, which is catalyzed by specific oxidosqualene cyclases (OSCs). Molecular characterizations of these genes involved in sterol biosynthesis have been intensively studied in many plant species, such as *Arabidopsis thaliana*, and *Solanum lycopersicum* [14,15,16,17,18]. In addition, although the three pathway enzymes, SQS [19], SE [20] and β-amyrin synthase (an OSC enzyme) [21], have been characterized in *E. tirucalli*, the comprehensive understanding of sterol biosynthesis in *E. tirucalli* is still limited.

**Figure 1:**
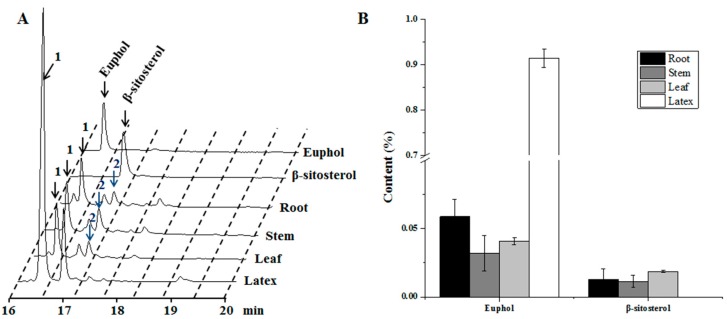
Phytochemical analysis of different *Euphorbia tirucalli* tissues. (**A**) GC-traces of the extracts from root, stem, leaf, and latex samples. In the figure: peak1, euphol; peak 2, β-sitosterol; (**B**) The concentrations of euphol and β-sitosterol in different fresh tissues. GC: Gas-Chromatograpy.

Given the fact that many types of plant metabolites are tissue-specifically biosynthesized, transcriptomic comparison between plant tissues is an effective approach to discover the key genes involved in various metabolic pathways [22,23]. In this study, tissue-specificity of sterol biosynthesis was also exhibited in *E. tirucalli* (Figure 1). With the aim of uncovering the candidate genes involved in sterol biosynthesis in *E. tirucalli*, a transcriptome was integrated from different tissues including root, leaf, stem and latex. Extensive bioinformatics analysis of this transcriptome further provided a molecular explication for the tissue-specificity of sterol synthesis in *E. tirucalli*, especially for the biosynthesis of euphol in latex.

## 2. Materials and Methods

### 2.1. Plant Material and RNA Extraction

*E. tirucalli* plants were cultivated in a greenhouse (Wuhan Botanical Garden, Chinese Academy of Sciences, Wuhan, China) under natural sunlight conditions with the temperature set at 25–28 °C and the relative humidity maintained at 35–55%. The materials (root, stem, leaf and latex) were collected from three independent plants. Exuded latex from the cut stem of *E. tirucalli* was rapidly mixed with three volumes of extraction buffer (100 mM phosphate buffer (pH 7.0), 10 mM EDTA (pH 8.0), and 0.1% (*v*/*v*) β-mercaptoethanol), immediately frozen in liquid nitrogen, and stored at −80 °C until use.

Total RNA was isolated from each tissue using the EASYspin plant RNA extraction kit (Aidlab Biotechnologies, Co., Ltd., Beijing, China) and further digested with DNase I (Thermo Fisher Scientific Inc., Wilmington, DE, USA) to remove genomic DNA contamination. Then, the RNA was visualized by electrophoresis on 1% agarose gel, and its integrity and quantity were determined using an Agilent 2100 Bioanalyzer (Agilent Technologies, Palo Alto, CA, USA).

### 2.2. Phytochemical Analysis

For the metabolite analysis of root, stem and leaf samples, 100 mg (fresh weight) of the sample was powdered in liquid nitrogen prior to extraction. For the extraction from latex, 100 mg of the latex was carefully sampled. The materials (root, stem, leaf and latex) were collected from three independent plants for metabolite analysis, which were the identical individuals for RNA extraction. The powdered plant materials and latex were suspended in 1 mL of ethyl acetate: ethanol (4:1, *v*/*v*) and then subjected to ultra-sonication for 60 min. The solvent extracts were then evaporated and derivatized with *N,O*-Bis (trimethylsilyl) trifluoroacetamide (BSTFA, Sigma-Aldrich, St. Louis, MO, USA) prior to Gas Chromatography-Mass Spectrometer (GC-MS) analysis. The GC-MS analysis was performed on an Agilent Technologies 5975C instrument with the carrier gas at a flow rate of 0.8 mL/min. Two microliters of each sample were injected into a GC column (HP-5ms column, 30 m × 0.25 mm × 0.25 μm, Agilent Technologies, Palo Alto, CA, USA) with an injector temperature of 250 °C. The initial oven temperature was 80 °C and held for 2 min. Then, it was heated to 310 °C at a rate of 20 °C/min and maintained for 15 min. The compound mass was detected in SIM-scan mode with electric ionization, and the MS range was set from 50 to 600 *m*/*z*. The standard curve method was used to determine the concentration of sterol compounds in fresh tissues of three independent replicates, with euphol and β-sitosterol as authentic standards.

### 2.3. Transcriptome Sequencing and de Novo Assembly

The well-qualified total RNA extracted from the root, stem and leaf samples was mixed in equal amounts into a single pool and then sent to Novogene Bioinformatics Technology Co., Ltd. (Beijing, China) for cDNA library construction and RNA-sequencing using the Illumina HiSeq X Ten platform (Illumina, San Diego, CA, USA) according to the manufacturer’s protocol. The paired-end reads were generated with a length of 150 bp for each read. The raw sequenced data have been submitted to the National Center for Biotechnology Information (NCBI) database with the accession number SRR6282416 for the root, SRR6282415 for the stem and SRR6282414 for the leaf.

Although we tried our best to extract the total RNA from latex many times, the RNA quality could not meet the requirements for RNA sequencing. However, latex transcriptome data (SRP073643) obtained by Illumina sequencing are available from the NCBI database [24]. Hence, the raw reads derived from the three samples (root, stem, and leaf) were pooled with the published *E. tirucalli* latex sequence data. After discarding low quantity reads from the raw data, these clean short reads were de novo assembled into transcript contigs or unigenes using Trinity software [25] with default parameters.

### 2.4. Functional Annotation

The function of unigenes was annotated by a blastx search (*e*-cut off value < 10^−5^) against four protein databases, including the NCBI non-redundant (Nr) protein database, Swiss-Prot protein database, Clusters of Orthologous Groups of protein database (COG), and Kyoto Encyclopedia of Genes and Genomes databases (KEGG) [26]. For the gene ontology (GO) annotation of the unigenes, the BLAST2GO program (https://www.blast2go.com/) was employed.

### 2.5. Differential Expression Analysis of Unigenes

Transcript abundance of the unigenes in each tissue sample was calculated and normalized using the FPKM (Fragments Per Kilobase Million) method [27]. Differentially expressed gene (DEG) analysis was performed using the DEGseq R package with a threshold of |log_2_ (fold change)| > 1 and corrected *p*-value < 0.05 [28]. GO enrichment analysis was performed by mapping the DEGs to the GO database and the gene numbers from each GO terms were calculated as compared to the genomic background [29]. Visualization of the clusters of the DEGs expression pattern was performed using the Multi Experiment Viewer (MEV; ver. 4.9).

### 2.6. Real-Time PCR Analysis

After digestion with DNase I, 2 µg of total RNA was converted to first-strand cDNA using Revert Aid Reverse Transcriptase (Fermentas, Thermo Fisher Scientific Inc., Wilmington, DE, USA). Real-time quantitative reverse transcription PCRs (qRT-PCRs) were performed using the ABI 7500 Real-Time PCR Detection System (Applied Biosystems, Foster City, CA, USA) with FastStart Universal SYBR Green Master Mix (Roche Diagnostics, Mannheim, Germany) as the fluorescent nucleotide dye. All primers were designed using Primer Premier ver. 5.0 software and are listed in Table S5.

## 3. Results

### 3.1. Tissue-Specificity for the Biosynthesis of Sterol Compounds in E. tirucalli

Euphol and β-sitosterol are two potential bioactive compounds in *E. tirucalli*. Phytochemical analysis of the two compounds in different tissues indicated that euphol was tissue-specifically biosynthesized in latex, and only small amount of euphol was detected in the other three tissues (Figure 1). Notably, accumulation of euphol in these tissues was more than 10-fold lower than that in latex. On the other hand, interestingly, we did not detect the occurrence of β-sitosterol in latex, although it was found in the root, stem and leaf tissues (Figure 1). We also calculated the relative ratio of the peak area of euphol and β-sitosterol to the total peak area presented in the GC-MS total ion chromatogram (TIC) profiles for the four tissues (Figure S1). The peak area of euphol accounted for 47.5% of the total peak area in the latex, which was much higher than that from the other tissues. However, the peak area of β-sitosterol only accounted for 0.9–1.75% in the TIC profiles of the root, stem and leaf tissues. The identities of the two compounds were also confirmed by aligning their retention times and mass fragmentation patterns with those of their respective authentic standards (Figure S2). These results suggest that the sterol compounds were biosynthesized in a strict tissue-specific manner in *E. tirucalli*.

### 3.2. Construction of the E. tirucalli Transcriptome Integrated from Different Tissues Including the Latex

The raw reads derived from the three samples (root, stem, and leaf) were pooled with the available *E. tirucalli* latex sequence data (SRP073643). After removing the adapter sequences and low-quality reads, a total of 21.76 Gb clean data derived from the root, stem and leaf tissues was obtained and then *de novo* assembled with the latex clean reads. The *E. tirucalli* transcriptome integrated from the root, stem, leaf and latex was finally constructed, and a total of 91,619 unigenes were generated from the transcriptome. The average length of the unigenes was 1351 bp with an N50 length of 2124 bp (Table 1). Sequence length distribution analysis showed that approximately 43.7% of the unigenes had a length of more than 1.0 Kb (Figure S3). These results suggested that the qualities of the sequencing and assembling were good enough for further bioinformatics analysis.

### 3.3. Functional Annotation and Classification of Unigenes

The putative functions of the assembled unigenes were annotated by blast searching against several public protein databases, of which 51.08% (46,804 unigenes) of the unigenes were successfully annotated against the Nr database (Table 2). The species distribution analysis revealed that the *E. tirucalli* unigenes showed the highest homology to genes from the Euphorbiaceae plant family, with 44.8% of the unigenes related to *Jatropha curcas*, and 30.1% of the unigenes ascribed to *Ricinus communis* (Figure S4).

**Figure 2:**
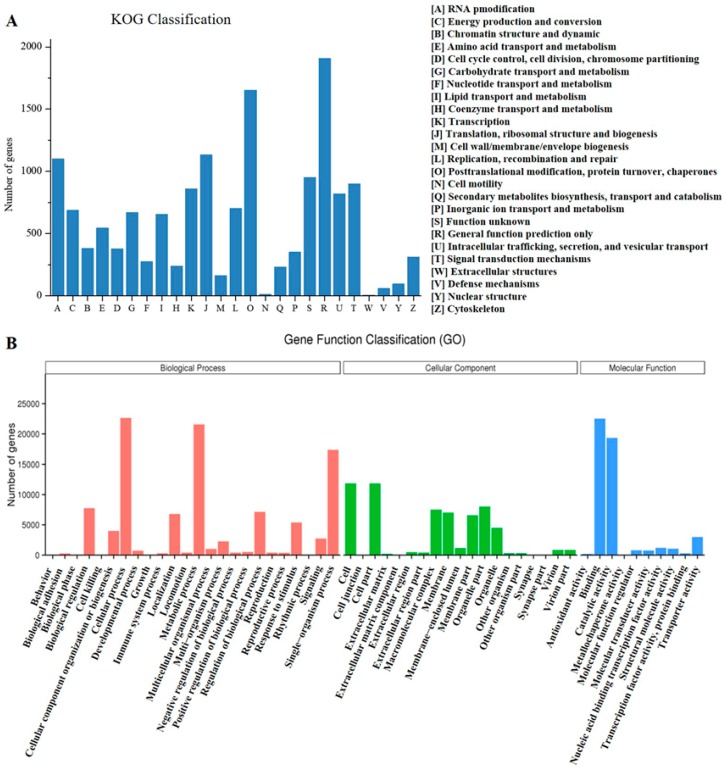
Classification of the function of the *E. tirucalli* unigenes based on the KOG (**A**) and GO (**B**) analysis.

Based on the KOG (EuKaryotic Orthologous Groups) database, 14.50% (13,292 unigenes) of the unigenes were categorized into 25 different functional groups (Figure 2A). Among them, the cluster of “General function prediction only” represented the largest group (1864 unigenes, 14.02% of KOG hits), followed by the group of “Post-translational modification, protein turnover, chaperones” (1653 unigenes, 12.44%). With respect to the attention of this study, we found that there were 253 unigenes associated with the group of “Secondary metabolite biosynthesis, transport and catabolism”. To further elucidate the function of these *E. tirucalli* unigenes, GO (Gene Ontology) analysis was also performed by using the Blast2 GO program [30]. A total of 38,652 unigenes (42.18% of the total unigenes) were successfully assigned to GO functional classification and grouped into three main categories (“Biological process”, “Cellular component”, and “Molecular function”). Among them, “Biological process”, “Cellular process” (22,862 unigenes, 59.14%) and “Metabolic process” (21,734 unigenes, 56.22%) represented the two most abundant GO terms. In reference to the KEGG database, 19.04% (17,447 unigenes) of the unigenes were mapped into 130 pathways based on five types of KEGG classifications: “Cellular processes”, “Environmental information processing”, “Genetic information processing”, “Metabolism”, and “Organismal systems” (Figure S5). Most of them were located in the pathway of “Carbohydrate metabolism” (1408 unigenes, 8.07% of KEGG hits), followed by “Translation” (1360 unigenes, 7.80%) (Table S1). In addition, there were 447 unigenes classified in the group of “Metabolism of terpenoids and polyketides”. Among them, 106 unigenes were annotated to play roles in “Terpenoid backbone biosynthesis” (ko00900) and the other 90 unigenes were involved in “Sesquiterpenoid and triterpenoid biosynthesis” (ko00909).

### 3.4. Gene Expression Analysis across Different Tissues

**Figure 3:**
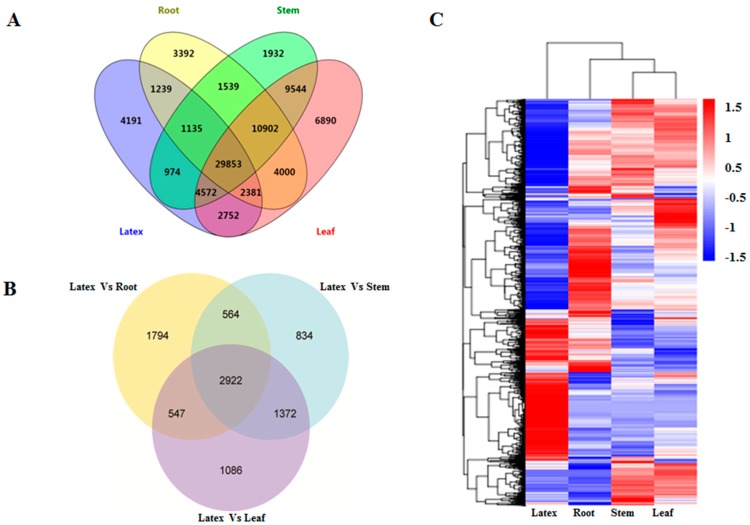
Expression analysis of the *E. tirucalli* unigenes. (**A**) Venn diagram analysis for the unigenes with their Fragments Per Kilobase Million (FPKM) values above 0.3 in different tissues; (**B**) Differentially Expressed Genes (DEG) analysis between tissues: latex vs. root, latex vs. stem and latex vs. leaf; (**C**) Heatmap analysis showing the expression pattern of DEGs in different tissues.

In order to investigate the DEGs among the four *E. tirucalli* tissue samples, the FPKM method was employed, and the unigenes with a significant level of FPKM value above 0.3 were selected. A total of 54,441 unigenes from the root, 60,451 from the stem, 70,894 from the leaf, and 47,097 from the latex were found to have higher FPKM values above 0.3 (Table S2). Among them, 29,853 unigenes were expressed in all of the four tissues, while 3392, 1932, 6890, and 4191 unigenes were specifically expressed in the root, stem, leaf, and latex, respectively (Figure 3A). The pairwise comparison of DEGs between latex and three other tissues revealed that the numbers of DEGs between latex and leaf, latex and root, and latex and stem were 5927, 5827, and 5692, respectively. Among all the DEGs, 2922 of them were found to be overlapped across all three sets of pairwise comparisons as shown in the Venn diagram (Figure 3B). Moreover, the cluster analysis of these DEGs revealed that the gene expression pattern in latex was distinctly different from that in the other three tissues, suggesting that the transcriptional regulations of DEGs for their functions were unique in the latex (Figure 3C). The DEGs were further applied for the GO enrichment analysis and KEGG pathway enrichment analysis (Table 3). Based on the KEGG analysis, a large portion of DEGs, which exhibited higher expression levels in the latex compared to the other tissues, were found to be associated with several secondary metabolite pathways, such as, the “Sesquiterpenoid and triterpenoid biosynthesis” (ko00909), “Terpenoid backbone biosynthesis” (ko00900), and “Steroid biosynthesis” (ko00100). On the other hand, the unigenes relevant to photosynthesis were significantly down-regulated in latex compared to the leaf and stem tissues, which could be explained as latex is definitely not a structure required for photosynthesis.

To verify the reliability of DEGs identified by the FPKM method, 18 DEGs involved in the sterol biosynthesis were selected and their gene expression levels in the root, stem and leaf tissues were monitored by RT-qPCR analysis. As shown in Figure S6, the gene expression patterns for 15 DEGs (except for *MVD*, *HDR*, and *EtOSC2*) matched the results calculated by the FPKM method based on the transcriptome, suggesting that the FPKM method was reliable for gene expression analysis.

### 3.5. Transcript Abundance of the Upstream Pathway Genes Supported the Tissue Specificity for Euphol Biosynthesis in E. tirucalli

**Figure 4:**
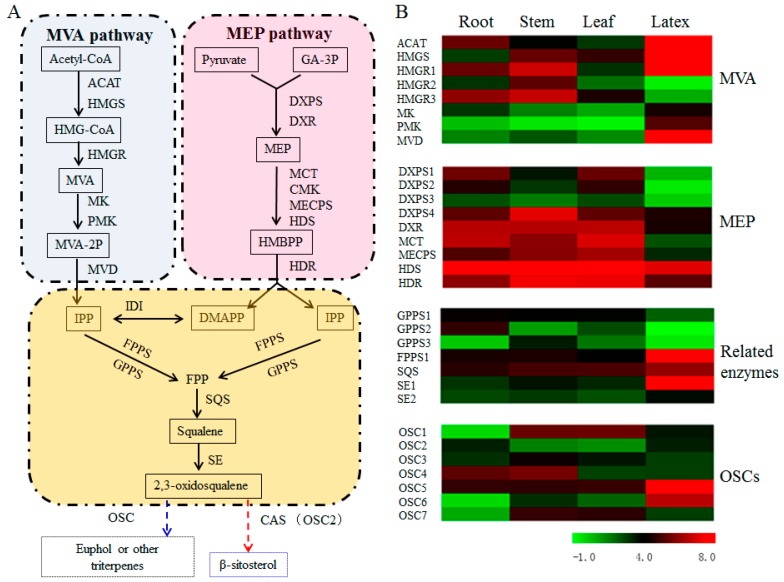
Expression analysis of the unigenes in the steps to euphol and β-sitosterol across the four *E. tirucalli* tissues. (**A**) Proposed biosynthetic pathways for euphol and β-sitosterol; (**B**) Heatmap analysis showing the expression patterns of DEGs in different tissues. The transcript abundances of relevant genes were analyzed by the FPKM method. The green color indicates the low transcriptional level, and the red color indicates the high transcriptional level. In the figure: ACAT, acetyl-CoA acetyltransferase; HMGS, hydroxymethylglutaryl-CoA synthase; HMGR, hydroxymethylglutaryl-CoA reductase; MK, mevalonate kinase; PMK, phosphomevalonate kinase; MVD, mevalonate diphosphate decarboxylase; DXPS, 1-deoxy-D-xylulose-5-phosphate synthase; DXR, 1-deoxy-D-xylulose 5-phosphate reductoisomerase; MCT, 2-C-methyl-D-erythritol 4-phosphate cytidylyltransferase; CMK, 4-diphosphocytidyl-2-C-methyl-D-erythritol kinase; MECPS, 2-C-methyl-D-erythritol 2,4-cyclodiphosphate synthase; HDS, (E)-4-hydroxy-3-methylbut-2-enyl-diphosphate synthase; HDR, 4-hydroxy-3-methylbut-2-enyl diphosphate reductase; IDI, isopentenyl-diphosphate delta-isomerase; GPPS, geranyl diphosphate synthase; FPPS, farnesyl diphosphate synthase; SQS, squalene synthase; SE, squalene epoxidase; and OSC, oxidosqualene cyclase.

The biosynthesis of plant sterols stems from the isoprenoid metabolism via MVA or MEP pathway. A total of 10 unigenes were identified to encode six enzymes in MVA pathway and the other 12 unigenes were inferred to encode seven enzymes in MEP pathway (Figure 4A and Table S3). As shown in Figure 4B, the transcript abundances of the three rate-limiting enzymes in the MVA pathway, namely acetyl-CoA acetyltransferase (ACAT), hydroxymethylglutaryl-CoA synthase (HMGS) and hydroxymethylglutaryl-CoA reductase (HMGR), were all increased in the latex. Moreover, the cluster-16161.34222 for FPPS, the cluster-16161.42353 for SQS, and the cluster-16161.34024/65659 for SE, which were involved in the pathway from IPP to 2,3-oxidosqualene, were all up-regulated in latex compared to three other tissues, with GPPS encoding gene as an exception which displayed the down-regulation in the latex (Figure 4B). Therefore, the increased transcript levels of upstream pathway genes might contribute to the higher-level accumulation of euphol in latex. It is well known that plant sterols are mainly derived from the MVA pathway rather than the MEP route [31]. In this study, we also found that a majority of the MEP pathway genes were down-regulated in the latex, verifying that the biosynthesis of euphol was mainly via the MVA-independent route rather than the MEP pathway.

### 3.6. Identification of OSCs in the Transcriptome of E. tirucalli

**Figure 5:**
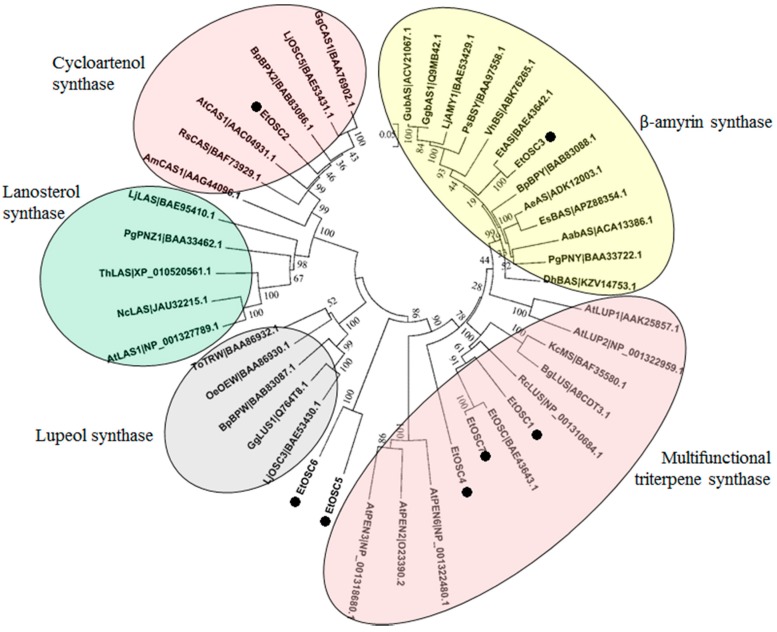
Phylogenetic analysis of the putative OSCs identified in the *E. tirucalli* transcriptome. The OSCs protein sequences were aligned by ClustalX software and the phylogenetic tree was constructed by the neighbor-joining method using MEGA ver. 5.0 software with 1000 bootstraps. In the figure: LjLAS: BAE95410.1, *Lotus japonicus*; PgPNZ1: BAA33462.1, *Panax ginseng*; AtLAS1: NP_001327789.1, *A. thaliana*; NcLAS: JAU32215.1, *Noccaea caerulescens*; ThLAS: XP_010520561.1, *Tarenaya hassleriana*; RsCAS: BAF73929.1, *Rhizophora stylosa*; AmCAS1: AAG44096.1, *Abies magnifica*; BpBPX2: BAB83086.1, *Betula platyphylla*; LjOSC5: BAE53431.1, *L. japonicus*; GgCAS1: BAA76902.1, *Glycyrrhiza glabra*; AtCAS1: AAC04931.1, *A. thaliana*; KcMS: BAF35580.1, *Kandelia candel*; BpBPY: BAB83088.1, *B. platyphylla*; GubAS: ACV21067.1, *Glycyrrhiza uralensis*; AeAS: ADK12003.1, *Aralia elata*; EtOSC: BAE43643.1, *E. tirucalli*; EtAS: BAE43642.1, *E. tirucalli*; DhBAS: KZV14753.1, *Dorcoceras hygrometricum*; VhBS: ABK76265.1, *Vaccaria hispanica*; PsBSY: BAA97558.1, *Pisum sativum*; AabAS: ACA13386.1, *Artemisia annua*; EsBAS: APZ88354.1, *Eleutherococcus senticosus*; GgbAS1: Q9MB42.1, *G. glabra*; LjAMY1: BAE53429.1, *L. japonicus*; PgPNY: BAA33722.1, *P. ginseng*; RcLUS: NP_001310684.1, *R. communis*; AtLUP1: AAK25857.1, *A. thaliana*; BpBPW: BAB83087.1, *B. platyphylla*; BgLUS: A8CDT3.1, *Bruguiera gymnorhiza*; LjOSC3: BAE53430.1, *L. japonicus*; OeOEW: BAA86930.1, *Olea europaea*; ToTRW: BAA86932.1, *Taraxacum officinale*; GgLUS1: Q764T8.1, *G. glabra*; AtLUP2: NP_001322959.1, *A. thaliana*; AtPEN6: NP_001322480.1, *A. thaliana*; AtPEN2: O23390.2, *A. thaliana*; and AtPEN3: NP_001318680.1, *A. thaliana*.

It is well known that the cyclisation of 2,3-oxidosqualene catalyzed by OSCs is a critical branching point for sterol and triterpenoid biosynthesis [32]. In the present study, seven putative *OSCs* (termed as *EtOSCs*) were identified in the *E. tirucalli* transcriptome (Table S4). These seven EtOSCs were subjected to the phylogenetic analysis together with many previously well-characterized OSCs. As shown in Figure 5, EtOSC1, EtOSC4, and EtOSC7 are clustered in the same sub-clade with multifunctional triterpene synthases, including AtLUP1 and AtLUP2, which cyclize 2,3-oxidosqualene to mainly yield lupeol with some minor by-products. EtOSC2 shows the 82% amino acid identity with a cycloartenol synthase (CAS) AtCAS1 from *A. thaliana*, indicating that EtOSC2 is a CAS in *E. tirucalli*. EtOSC3, previously named as EtAS [21], is a β-amyrin synthase. EtOSC5 and EtOSC6 show relatively closer relationship with AtPEN3 (At5g36150) from *A. thaliana*. It has been reported that the cyclisation of 2,3-oxidosqualene by AtPEN3 predominantly produces tirucalla-7,24-dien-3-ol with several minor side products (i.e., 6% of tirucallol) [33,34]. Both euphol and tirucallol are the isomers of tirucalla-7,24-dien-3-ol. As shown in Figure 4B, EtOSC5 and EtOSC6 were highly expressed in latex compared to the other tissues. These data tempted us to speculate that EtOSC5 and EtOSC6 might be the candidate OSC enzymes for euphol synthesis in *E. tirucalli.*

## 4. Discussion

Sterols in the *E. tirucalli* latex, including euphol and β-sitosterol, are thought to be related to various medicinal properties of this plant in previous phytochemical studies [35]. Interestingly, in this study, a contrasting tissue-specificity was revealed for euphol and β-sitosterol in *E. tirucalli*, as euphol was highly accumulated in the latex with the absence of β-sitosterol. This phytochemical data indicated that the expressions of genes involved in sterol biosynthesis were distinctly regulated in different *E. tirucalli* tissues. To decipher the mechanism underlying the tissue-specific biosynthesis of sterols in *E. tirucalli*, a transcriptome database integrated from the root, stem, leaf and latex of the plant was constructed, and the genome-wide expression was extensively analyzed. With respect to the upstream pathway genes, most of the MVA pathway genes were up-regulated in latex compared to the other tissues, which was consistent with the high-level accumulation pattern of euphol in latex. On the other hand, the expression levels of most MEP pathway genes were significantly down-regulated in the latex compared to the other tissues. These gene expression data agreed with the well-accepted conclusion that the biosynthesis of triterpenoids or plant sterols is mainly derived from the MVA pathway rather than the MEP pathway [36]. In the pathway after IPP, the transcript abundance of most of the pathway genes, such as those encoding FPPS, SQS, and SE, was also significantly higher in latex than that in the other tissues, which further demonstrated that the tissue-specificity for euphol biosynthesis was likely due to the transcriptional regulation of the pathway genes.

In fact, the biosynthesis of euphol and β-sitosterol shares the common intermediate 2,3-oxidosqualene, which is then channeled to β-sitosterol and euphol by CAS and euphol synthase, respectively [37]. Although lanosterol was recently reported to be a substrate for the biosynthesis of β-sitosterol [16], none of the unigenes encoding lanosterol synthase was identified in the *E. tirucalli* transcriptome, suggesting that the biosynthesis of β-sitosterol in *E. tirucalli* was mediated by CAS. Based on the phylogenetic tree and transcript level analysis, EtOSC2 is likely the putative gene encoding CAS, while EtOSC5 and EtOSC6 seem to be the candidates for euphol synthase. Thus, these three unigenes are possibly specific to steroid biosynthesis. Although the accumulation of β-sitosterol was not detected in latex, the expressions of a great number of unigenes in the pathway converting to β-sitosterol were identified in the latex (Table S6). It was confusing that the expression of *EtOSC2* seemed to be inconsistent with the absence of β-sitosterol in the latex, since a notable expression of *EtOSC2* was observed in the latex. One possible reason is that β-sitosterol could be synthesized in the latex and subsequently conveyed to other tissue cells. Alternately, in the latex, the metabolic flux of the intermediate 2,3-oxidosqualene was diametrically channeled to the pathway of euphol biosynthesis, whereas the carbon flux towards β-sitosterol was tightly attenuated, leading β-sitosterol being at a detectable level. This assumption is based on the observation of much higher transcript levels of *EtOSC5* and *EtOSC6* than *EtOSC2* in the latex.

In conclusion, a tissue specific distribution of plant sterols in *E. tirucalli* was investigated. The comprehensive transcriptome database derived from the root, stem, leaf and latex of *E. tirucalli* was integrated, which formed a strong basis for identifying the putative genes involved in the biosynthesis of sterols or triterpenoids in this plant, and especially for exploiting these OSC candidate genes responsible for euphol biosynthesis. Further functional characterizations of these OSCs are urgently required to confirm their roles in triterpenoid biosynthesis. Moreover, a unique expression pattern of DEGs was also revealed in latex, which was distinctly different from that in the other tissues. The identified DEGs are valuable sources for further investigating the molecular mechanism underlying the tissue-specific regulation of sterol biosynthesis in plants.

## Figures and Tables

**Table 1:** Summary of sequencing and de novo assembling of the transcriptome of *E. tirucalli*.

Item	Sample	Number (n)	Total Nucleotides (G nt)	Valid Ratio (%)	GC (%)	Q30 (%)	N50 (bp)	Average Length (bp)
Raw reads	Latex	19,838,038	1.98	—	—		—	—
	Root	44,125,666	6.61	—	—		—	—
	Stem	54,363,252	8.15	—	—		—	—
	Leaf	54,091,184	8.11	—	—		—	—
Clean reads	Latex	19,590,464	1.95	98.48	42.71	96.34	—	—
	Root	42,271,760	6.34	95.92	43.15	89.50	—	—
	Stem	52,432,630	7.86	96.44	42.98	90.12	—	—
	Leaf	50,429,140	7.56	93.22	43.53	87.22	—	—
Total	Unigenes	91,619	0.12	—	—	—	2099	1351

Valid ratio, the percentage of clean reads in raw reads; N50, the weighted median statistic, such that 50% of the bases in the assembly are contained in scaffolds (or contigs), of which the sizes are equal to or larger than this value. Q30: the percentage of the bases of Phred value above 30 (probability of incorrect bases less than 1/1000) in the original data accounts.

**Table 2:** Statistics of annotations for assembled unigenes in the transcriptome of *E. tirucalli*.

Database	Number of Unigenes	Percentage (%) ^b^
Nr ^a^	46,804	51.08
SwissProt	36,923	40.30
KOG	13,292	14.50
GO	38,652	42.18
KEGG	17,447	19.04
Annotated in all databases	7835	8.55
Annotated in at least one database	52,448	57.24

^a^ Nr: NCBI non-redundant protein database; ^b^ Percentage of annotated unigenes in the total 91,619 assembled unigenes. KOG: EuKaryotic Orthologous Groups (database); GO: Gene Ontology (database); KEGG: Kyoto Encyclopedia of Genes and Genomes.

**Table 3:** KEGG pathway enrichment analysis of DEGs between latex and the other tissues.

	Pathway Term	Number	Pathway Term	Number	Pathway Term	Number
	Latex vs. Leaf		Latex vs. Root		Latex vs. Stem	
Up-regulated DEGs in latex compared to other tissues	Sesquiterpenoid and triterpenoid biosynthesis	19	Sesquiterpenoid and triterpenoid biosynthesis	19	Sesquiterpenoid and triterpenoid biosynthesis	17
	Endocytosis	32	Photosynthesis—antenna proteins	11	Terpenoid backbone biosynthesis	15
	Regulation of autophagy	15	Photosynthesis	14	Peroxisome	25
	Terpenoid backbone biosynthesis	15	Galactose metabolism	17	Endocytosis	29
	Galactose metabolism	17	Regulation of autophagy	14	Galactose metabolism	15
	Steroid biosynthesis	11	Butanoate metabolism	8	Synthesis and degradation of ketone bodies	3
	Ether lipid metabolism	8	Pyruvate metabolism	23	Regulation of autophagy	12
	Fatty acid biosynthesis	13	Terpenoid backbone biosynthesis	13	Protein processing in endoplasmic reticulum	33
	Synthesis and degradation of ketone bodies	3	Steroid biosynthesis	10	Fatty acid biosynthesis	12
	Peroxisome	22	Peroxisome	22	Steroid biosynthesis	9
Down-regulated DEGs in latex compared to other tissues	Photosynthesis	41	Ribosome	143	Photosynthesis	39
	Oxidative phosphorylation	54	Plant hormone signal transduction	57	Oxidative phosphorylation	56
	Photosynthesis - antenna proteins	14	Oxidative phosphorylation	43	Carbon fixation in photosynthetic organisms	34
	Porphyrin and chlorophyll metabolism	20	Citrate cycle (TCA cycle)	19	Photosynthesis - antenna proteins	14
	Carbon fixation in photosynthetic organisms	28	Carotenoid biosynthesis	15	Pentose phosphate pathway	28
	Carotenoid biosynthesis	17	Flavonoid biosynthesis	12	Porphyrin and chlorophyll metabolism	20
	Limonene and pinene degradation	15	Stilbenoid, diarylheptanoid and gingerol biosynthesis	9	Glycolysis/Gluconeogenesis	46
	Ribosome	70	Phenylpropanoid biosynthesis	33	Ribosome	72
	Cell cycle—caulobacter	12	Spliceosome	46	Plant hormone signal transduction	47
	nitrogen metabolism	15	Cysteine and methionine metabolism	25	Glyoxylate and dicarboxylate metabolism	25

TCA (cycle): Tricarboxylic acid (cycle).

## Supplementary Materials

The following are available online at www.mdpi.com/2073-4425/9/1/38/s1. Figure S1. Relative ratio of the peak area of euphol and β-sitosterol to the total peak areas in the GC-MS TIC profiles for the four tissues of *E. tirucalli*; Figure S2: Alignments of the peaks (1 and 2) in the GC-MS spectrums of different tissue samples with their chemical standards; Figure S3: Sequence length distribution of unigenes in the transcriptome of *E. tirucalli*; Figure S4: Species distribution of the Nr annotated unigenes in the transcriptome of *E. tirucalli*; Figure S5: KEGG pathway analysis of unigenes in the transcriptome of *E. tirucalli*; Figure S6: RT-qPCR analysis of the gene expression pattern of the DEGs identified by the FPKM method; Table S1: KEGG annotation of DEGs in the transcriptome of *E. tirucalli*; Table S2: Specific unigenes with FPKM values above 0.3; Table S3: Specific unigenes involved in triterpene backbone biosynthesis; Table S4: Specific unigenes encoding OSCs involved in terpenoid biosynthesis; Table S5: The qRT-PCR primers used in this study, Table S6: Specific unigenes involved in β-sitosterol biosynthesis.
