# Supplementary material for: Comparative Transcriptome Analysis Identifies Putative Genes Involved in Steroid Biosynthesis in Euphorbia tirucalli
Source: Genes (Basel). 2018 Jan 15;9(1):38. doi: 10.3390/genes9010038 (PMC5793189; doi:10.3390/genes9010038)
Supplement: Supplementary file 1 [file genes-09-00038-s001.zip › supplementary data/genes-248619-Supplementary material.docx]

*Additional files*

Comparative Transcriptome Analysis Identifies Putative Genes Involved in the Steroid Biosynthesis in *Euphorbia tirucalli*

**Weibo Qiao^1, 2^, Changfu Li^1^,** **Isidore Mosongo^1, 2^, Qin Liang^1, 2^, Mengdi Liu^1, 2^, Xin Wang^1^***

^1^ CAS Key Laboratory of Plant Germplasm Enhancement and Specialty Agriculture, Wuhan Botanical Garden, Chinese Academy of Sciences, Wuhan, 430074, China;

^2^ University of Chinese Academy of Sciences, Beijing, 100049, China

***** Correspondence: wangxin@wbgcas.cn

**Figure S1**


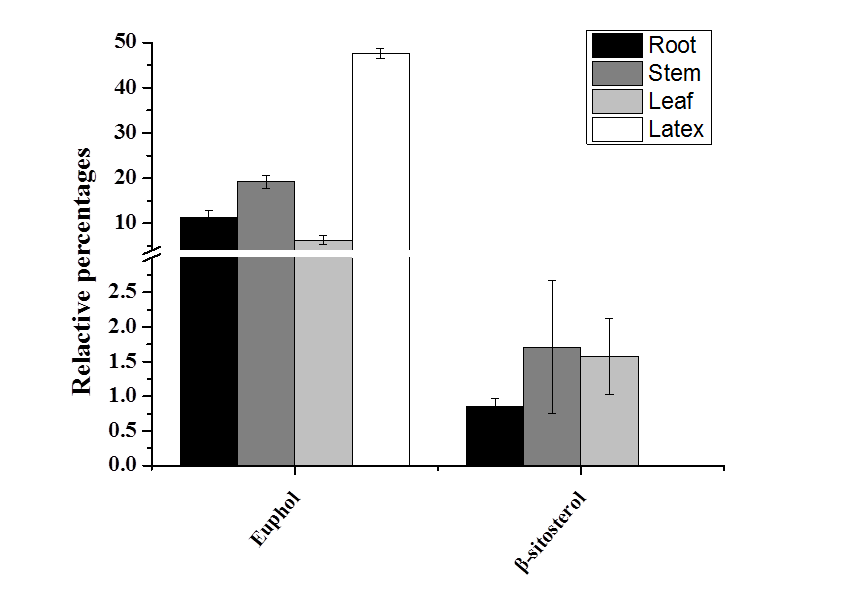


Figure S1. Relative percentages of the peak areas of euphol and β-sitosterol to the total peak areas in the GC –MS total ion chromatogram (TIC) profiles for different tissues of *E. tirucalli* (root, stem, leaf and latex). In the latex, the proportion of the euphol peak in the TIC profile was 47.5%, which was much higher than that from the other tissues. The β-sitosterol peak area accounted for 0.9-1.75% in the TIC profiles for the root, stem and leaf tissues, and no β-sitosterol was detected in the latex. For this part of the experiment, the plant materials were extracted with1 ml of ethyl acetate: ethanol (4:1, v/v) for 60 min, the solvent extracts were then evaporated and dissolved in 200 µl of methyl alcohol for the GC-MS analysis without the derivatization with BSTFA.

**Figure S2**


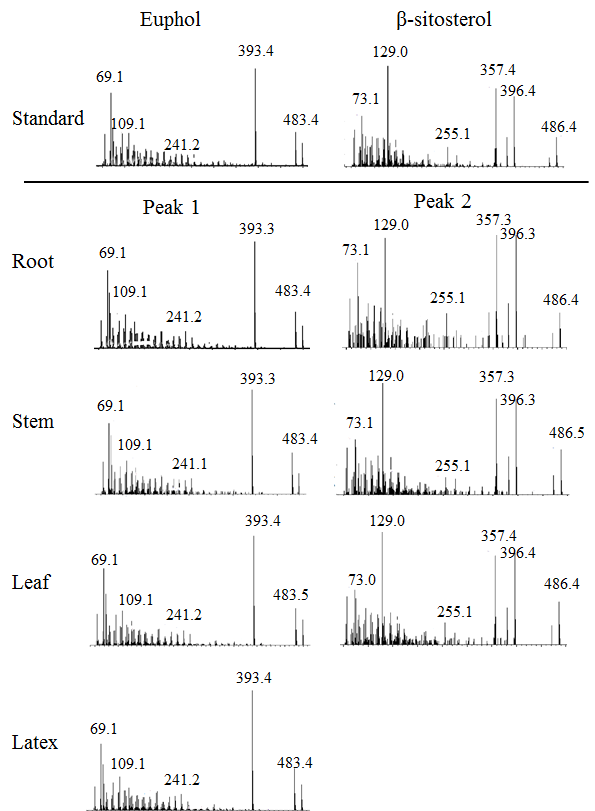


Figure S2. Alignment of the GC-MS spectrums of the sample peaks (1 and 2) from different tissues with their chemical standards. The peak 1 corresponded to euphol and the peak 2 was identified as β-sitosterol.

**Figure S3**


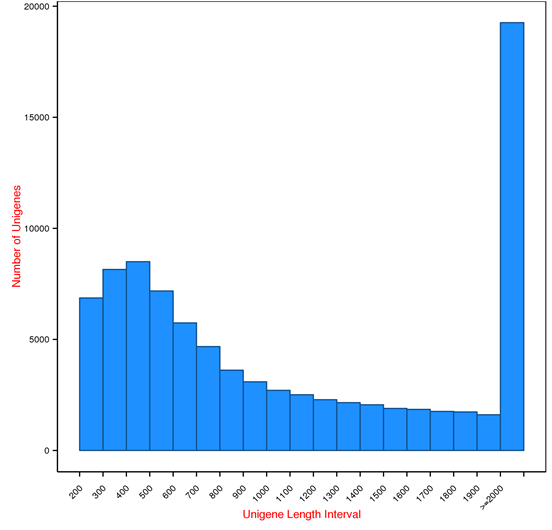


Figure S3 Length distribution of unigenes from the *E. tirucalli* transcriptome

**Figure S4**


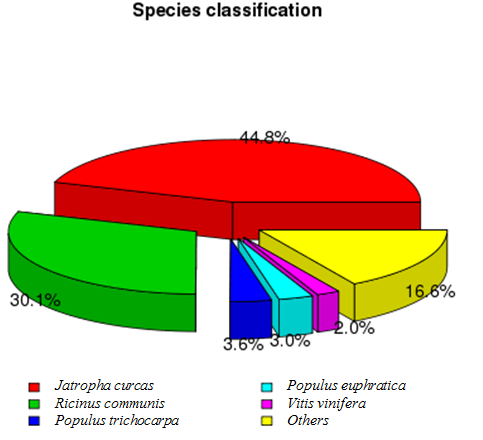


Figure S4 The species distribution of the Nr annotation from the *E. tirucalli* transcriptome. A Pie chart showed the similarity of unigenes of *E. tirucalli* with those from other species.

**Figure S5**


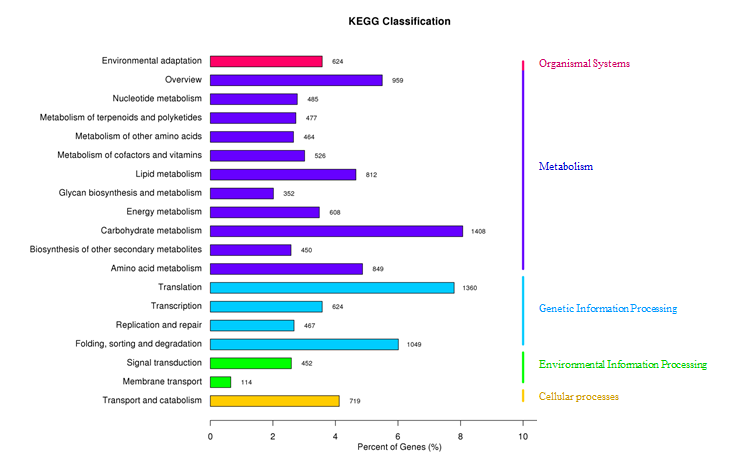


Figure S5 KEGG pathway analysis of unigenes from *E. tirucalli* transcriptome

**Figure S6**


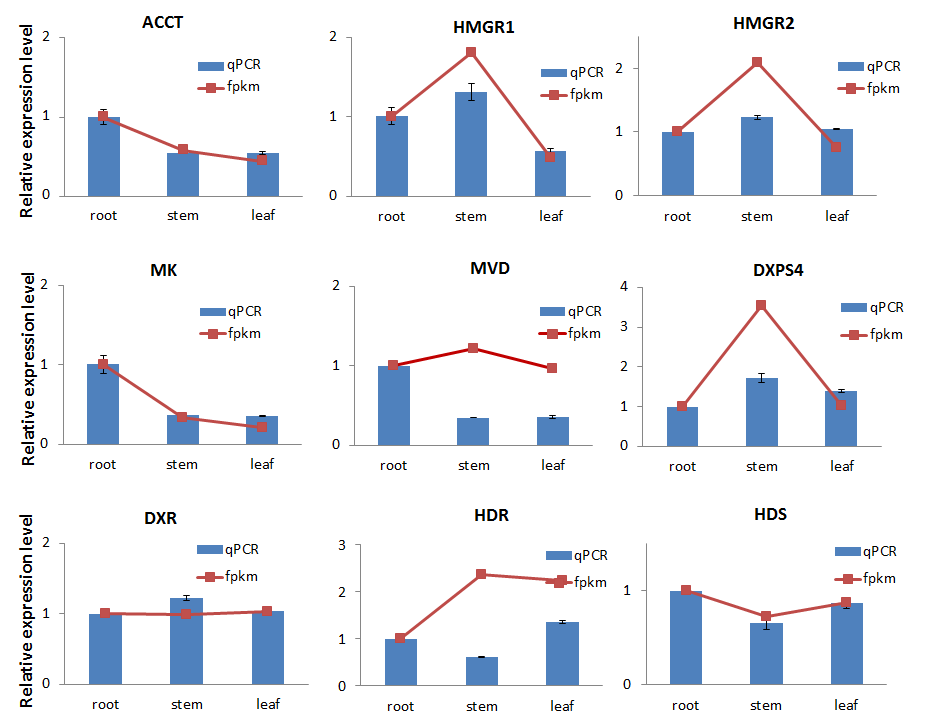


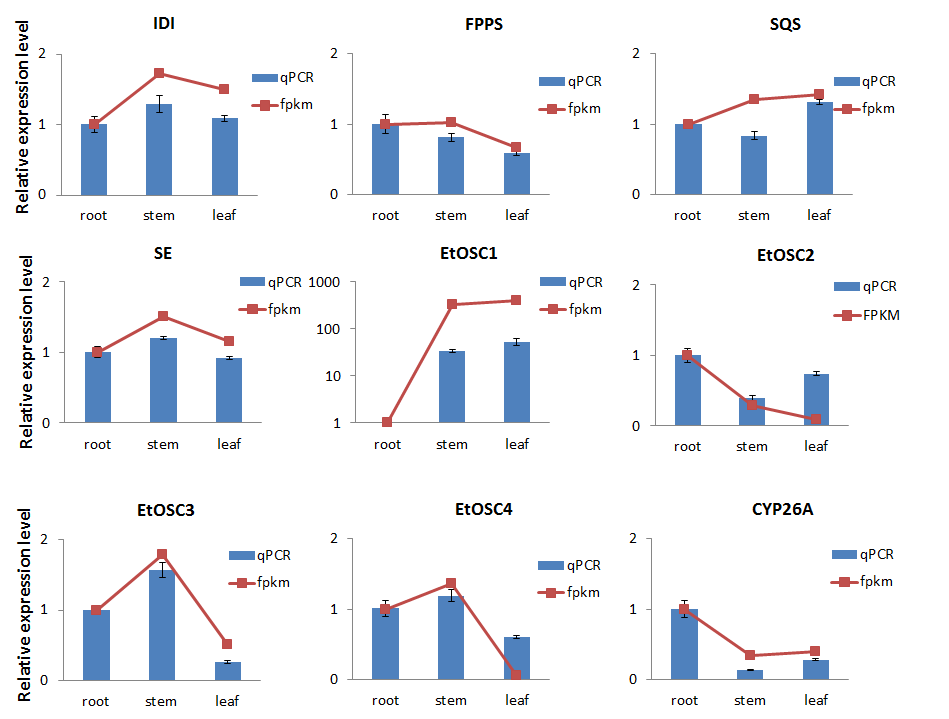


Figure S6 RT-qPCR confirmation of the tissue-specific gene expression of the selected DEGs identified by the transcriptome analysis using the FPKM method. Except for the MVD, HDR and SQS unigenes, the gene expression trends of all the rest tested DEGs were consistent between the transcriptome analysis and qPCR data, suggesting the reliability of the transcriptome analysis.
